# Data-driven discovery of the governing equations of dynamical systems via moving horizon optimization

**DOI:** 10.1038/s41598-022-13644-w

**Published:** 2022-07-12

**Authors:** Fernando Lejarza, Michael Baldea

**Affiliations:** 1 McKetta Department of Chemical Engineering, The University of Texas at Austin, 200 E Dean Keeton St, Austin, TX 78712-1589 USA; 2 Oden Institute for Computational Engineering and Sciences, The University of Texas at Austin, 201 E 24th St, Austin, TX 78712-1229 USA

**Keywords:** Applied mathematics, Computational science, Chemical engineering, Engineering

## Abstract

Discovering the governing laws underpinning physical and chemical phenomena entirely from data is a key step towards understanding and ultimately controlling systems in science and engineering. Noisy measurements and complex, highly nonlinear underlying dynamics hinder the identification of such governing laws. In this work, we introduce a machine learning framework rooted in moving horizon nonlinear optimization for identifying governing equations in the form of ordinary differential equations from noisy experimental data sets. Our approach evaluates sequential subsets of measurement data, and exploits statistical arguments to learn truly parsimonious governing equations from a large dictionary of basis functions. The proposed framework reduces gradient approximation errors by implicitly embedding an advanced numerical discretization scheme, which improves robustness to noise as well as to model stiffness. Canonical nonlinear dynamical system examples are used to demonstrate that our approach can accurately recover parsimonious governing laws under increasing levels of measurement noise, and outperform state of the art frameworks in the literature. Further, we consider a non-isothermal chemical reactor example to demonstrate that the proposed framework can cope with basis functions that have nonlinear (unknown) parameterizations.

## Introduction

Differential equation models play a critical role in describing the governing behavior of a variety of systems encountered in science and engineering. As minimal order expressions describing the system behavior, governing models are generalizable, readily interpretable, and have good extrapolation capabilities. Historically, the discovery and formulation of fundamental governing equations has been a relatively lengthy process, supported by careful experimentation and data collection using prototype experimental systems. Nonetheless, the data sets that have, through the history of science, supported the discovery of such fundamental natural laws may seem “small” by today’s standards. With decreasing costs of sensors, data storage systems, and computing hardware, immense quantities of data can be easily collected and efficiently stored. As a consequence, the applications of machine learning (ML) and artificial intelligence (AI) have witnessed meteoric growth in science and engineering. ML techniques can perform exceptionally well in regression and classification tasks, but the resulting models are most often black- or grey-box in nature, offering little physical insight, and extrapolate poorly to regimes beyond the range of the training data. Moreover, prediction accuracy typically comes at the cost of model complexity, which is at odds with the typically parsimonious nature of a system’s governing dynamics derived e.g. via first principles analysis.

Leveraging ML/AI frameworks to discover (as opposed to merely fit) the governing equations of physical systems from large amounts of data offers intriguing possibilities and remains an open field of research. Related recent efforts towards physically-constrained ML include physics-informed discovery strategies (of which a recent review can be found in^1^) combining first principles arguments with ML models, such as deep neural networks^2^. These models embed “informative priors”, e.g., mass and energy conservation laws, within ML architectures in order to improve their interpretability and reduce extrapolation error. Physics-informed neural networks (PINNs)^2^ are an example of such frameworks and have recently attracted significant attention in the literature, witnessing numerous extensions (e.g.,^3–6^). Nevertheless, it should be noted that these methods are mostly geared towards finding high fidelity data-driven solutions to partial differential equations (PDEs), as well as solving associated inverse problems by means of knowledge embedding, generally without emphasizing knowledge discovery.

Past work on automated data-driven discovery of governing equations is based on nonlinear regression strategies^7^. Initial efforts exploited symbolic regression^8^ and genetic programming algorithms^9^. However, the combinatorial nature of these approaches can render them computationally prohibitive, restricting their applicability to low-dimensional systems and to considering relatively small initial sets of candidate symbolic expressions (which inherently diminishes the success rate of identifying the true underlying system dynamics). Furthermore, symbolic regression strategies can be prone to overfitting, i.e., generating overly complex expressions in an attempt to decrease prediction error^8^. Later works^10,11^ combine deep learning architectures with established model fitting techniques (e.g., dimensional analysis, polynomial and symbolic regression), which result in improved performance relative to original symbolic regression schemes^8,12^. Despite their potential computational performance limitations, schemes based on genetic programming benefit from not requiring a complete library of basis functions (i.e., new terms can be generated through crossover and mutation steps^13^).

In a different vein, sparse nonlinear regression techniques^14,15^ have been proposed to improve on the computational complexity of symbolic regression-based frameworks. Brunton et al.^16^ employed sequentially thresholded least-squares (STLSQ) and least absolute shrinkage and selection operator (LASSO), that is, $\ell _1$ regularized regression, for sparse identification of nonlinear dynamics (SINDy). These algorithms recover governing equations by identifying a small number of relevant nonlinear functions from within an *a priori* specified large set of candidate basis functions using state measurement data sets. Theoretical convergence properties have been established^17^, and the framework has been implemented as an open-source code^18^. Numerous extensions to SINDy have since been proposed, addressing a variety of classes of systems and problem settings including, e.g., PDEs^19,20^, unknown coordinate systems^21^, biological networks^22^, model predictive control^23^, isothermal chemical reactions^24^. A similar approach based on elastic net regression (i.e., a combination of both $\ell _1$ and $\ell _2$ regularizations) was introduced in^25^, but resulted in less parsimonious equations relative to e.g. LASSO regression. In a related effort^26^, the selection of basis functions was performed via mixed-integer nonlinear programming, with a view towards identifying low-order surrogate representations of nonlinear algebraic models.

Recent efforts have focused on extending the SINDy concept to cope with corrupted data sets, as well as dynamical systems of higher complexity. For example, to overcome the instability associated with computing derivatives directly from noisy data, the integral (or weak) formulation of the system dynamics was used for systems of ODEs^27,28^ and (high-order) PDEs^29^. Similarly, Goyal et al.^30^ incorporated Runge-Kutta integration schemes within the dynamics discovery formulation to bypass direct estimation of derivatives from data and reduce gradient estimation errors relative to lower order methods. Extensions for automatically denoising measurement data, learning and parametrizing the associated noise distribution, and subsequently inferring the underlying parsimonious governing dynamical system were developed^31^. Similarly, Cao et al.^32^ proposed employing the Fourier transform of the original time series data to identify governing PDEs using the low frequency component of the signal, which significantly improves robustness to noise. Tran et al.^33^ proposed an $\ell _1$ minimization formulation and an alternating solution algorithm to discover chaotic systems when the data are corrupted with a large percentage of outliers. A later work^34^ introduced a unified framework leveraging non-convex sparsity promoting regularization functions (e.g., $\ell _0$ penalties) to detect and trim outliers while inferring the governing equations. The framework proposed in^34^ also allows for considering parametric dependencies in the basis functions, as well as physical constraints. Recently, Fasel et al.^35^ reported the use of bootstrap aggregation (“bagging”) by identifying an ensemble of SINDy models, which substantially improves robustness to noise while also allowing for uncertainty quantification and probabilistic forecasts. A related effort^36^ combined a weak formulation of differential equations with ensemble symbolic regression to identify fluid flow dynamics from a pool of variables and candidate models derived from general physical principles.

In this work, we propose a framework for discovering governing equations from noisy measurement data that is formulated via nonlinear dynamic optimization, a mathematical programming technique that offers substantially more flexibility than the previously cited examples. The proposed formulation aligns with ideas introduced in weak SINDy implementations^27–29^, in that it implicitly leverages advanced numerical integration (orthogonal collocation on finite elements^37^) to represent the underlying candidate governing equations, thus circumventing derivative estimation from data, reducing gradient approximation errors relative to lower order methods, and improving numerical stability. Further, we propose a method inspired by control theory, namely, moving horizon estimation^38^ and its counterpart, model predictive control^39^, to efficiently solve the aforementioned dynamic nonlinear program (DNLP). Moving horizon optimization strategies have been extensively used for reconstructing state values from noisy process measurements^38,40^, estimating model parameters from data^41^, or determining the optimal inputs for controlling dynamical systems whose equations have a *known* structure. Conversely, the methodology introduced here differs significantly from the moving horizon estimation canon in that the true structural form of the system dynamics is *unknown*, and sparsifying strategies are developed to recover parsimonious governing equations from a dictionary of candidate basis functions. The resulting sequence of discovered governing equations is aggregated (e.g., by taking the average of the estimated model coefficients and basis functions parameters), which is expected to further improve robustness to noise and allow for statistical characterization of the coefficient estimates in a similar sense as ensemble SINDy^35^.

Hence, relative to several of the previously cited works, the main contributions of the proposed framework are: (i) a general nonlinear programming-based optimization approach that can cope with parametric basis function libraries and can include domain knowledge-derived constraints, (ii) a moving horizon optimization framework that improves scalability and leverages statistical arguments to promote sparsity in the identified governing equations, and (iii) implicitly embedded discretization schemes that are stable and of high-order which improve robustness to noisy data, and are able to capture multiscale or stiff dynamics.

## Results

### Problem formulation

We consider dynamical systems governed by ordinary differential equations (ODEs) of the form:

1 $$\begin{aligned} \frac{d\mathbf{x }(t)}{dt} = \mathbf{f }(\mathbf{x }(t), \mathbf{u }(t)) \end{aligned}$$

where $\mathbf{x }(t) \in {\mathbb {R}}^{n_x}$ is the vector of states and $\mathbf{u }(t) \in {\mathbb {R}}^{n_u}$ is a vector of control (manipulated) inputs at time *t*, and the map $\mathbf{f }(\cdot ):{\mathbb {R}}^{n_x}\times {\mathbb {R}}^{n_u}\rightarrow {\mathbb {R}}^{n_x}$ represents the (nonlinear) dynamics of the system. The function $\mathbf{f }$ is *unknown* and is precisely what we attempt to infer from a given set of time-resolved measurement data. To that end, we collect a sequence of measurements $(\hat{\mathbf{x }}({\hat{t}}_j),\hat{\mathbf{u }}({\hat{t}}_j))$ of the state and input variables observed at sampling times ${\hat{t}}_0,\dots ,{\hat{t}}_m$, and assume that the derivative $\hat{\dot{\mathbf{x }}}({\hat{t}}_j)$ cannot be directly measured. The data are assumed to be contaminated with (Gaussian, zero-mean) measurement noise, and smoothing techniques and statistical tests are used to perform pre-processing of the training data set (See Methods). The resulting pre-processed data are denoted by $\tilde{\mathbf{x }}({\hat{t}}_j), \tilde{\mathbf{u }}({\hat{t}}_j) \; \forall j=0,\dots ,m$.

#### Remark 1

The proposed framework in the present form is intended to be used for analyzing systems whose dynamics are governed by ordinary differential equations. As it will be discussed at length subsequently, the ideas proposed herein can be readily extended to systems with spatial differential operators and to partial differential equations, an extension that will constitute a direction of future research.

**Figure 1:**
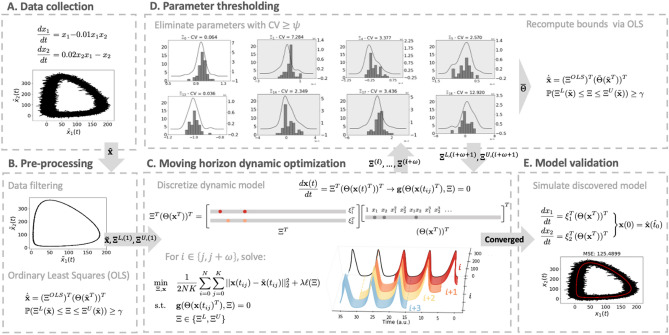
Summary of the present framework using the Lotka–Volterra system as an illustrative example, and for simplicity without considering control inputs $\mathbf{u }$ or parametric basis functions $\Theta (\mathbf{x }^T, \mathbf{c })$. (**A**) Noisy data $\hat{\mathbf{x }}$ are collected from physical experimentation or simulation. (**B**) The data are smoothed using filtering techniques and preliminary statistical analyses are performed to refine the library of basis functions and initialize the DNLP. (**C**) The dynamics are discretized and the DNLP is solved for the current window of data. (**D**) Every $\omega$ iterations of the moving horizon algorithm, coefficient thresholding is performed to eliminate basis functions having a coefficient of variation greater than a given tolerance (exemplified by histograms with grey background). (**E**) Once convergence is established the recovered equations are validated via simulation, by subsequently computing suitable regression error metrics, and by applying visualization techniques.

To discover the underlying governing equations, we consider a dictionary of $n_\theta$ candidate symbolic nonlinear basis functions denoted as $\Theta (\mathbf{x }^T,\mathbf{u }^T,\mathbf{c })$, where $\Theta (\cdot ): {\mathbb {R}}^{1\times n_x}\times {\mathbb {R}}^{1\times n_u} \rightarrow {\mathbb {R}}^{1\times n_\theta }$ and $\mathbf{c }\in {\mathbb {R}}^{n_c}$ is a vector that captures some unknown parametrization of the basis functions (e.g. let basis function *i* be $\theta _i$ such that $\theta _i(\mathbf{x }^T,\mathbf{u }^T) = \mathbf{u }^T\exp (-c_i\mathbf{x }^T)$). The dictionary is defined *a priori*, potentially leveraging some domain insights regarding the underlying system^25^. We assume that the governing equations can be expressed as a linear combination of the basis functions in this dictionary as:

2 $$\begin{aligned} \frac{d}{dt}\mathbf{x }(t) = \mathbf{f }(\mathbf{x }(t),\mathbf{u }(t)) = \Xi ^T \Theta (\mathbf{x }(t)^T,\mathbf{u }(t)^T,\mathbf{c })^T \end{aligned}$$

where $\Xi \in {\mathbb {R}}^{n_\theta \times n_x}$ is a matrix of coefficients whose columns are given by the sparse (i.e., most entries are zero) vectors $\pmb {\xi }_1, \dots ,\pmb {\xi }_{n_x}$. This sparse model structure is illustrated in Fig. 1C using the well-known two-dimensional Lotka–Volterra predator–prey model as an example. Further, we distinguish between *active* coefficients (i.e., $\xi _{i,j} \ne 0$ then basis function *j* is active for state variable *i* in the true governing equations), and *inactive* coefficients (i.e., $\xi _{i,j}=0$ in the true governing equations). Following the sparsity argument, the fundamental challenge of discovering the governing equations translates to identifying the (few) active coefficients associated with the basis functions that are truly present in $\mathbf{f }(\mathbf{x }(t),\mathbf{u }(t))$.

#### Remark 2

We note that the vast majority of existing frameworks concerned with using regression mechanisms to learn governing equations from data^16,25,27–29,35^ rely on solving a linear optimization problem to estimate the unknown coefficients $\Xi$, and cannot cope with parameterized libraries of basis functions that require estimating coefficients $\Xi$ and parameters $\mathbf{c }$ in function $\Theta (\cdot )$ in (2), simultaneously. Equivalently, the existing frameworks assume implicitly or explicitly that the dynamics in (1) can be expressed as $\Xi ^T\Theta (\mathbf{x } (t)^T,\mathbf{u } (t)^T)$, i.e., a function that is linear with respect to all unknown parameters and coefficients. The ability to handle the structural form in (2) represents a key advantage of the framework proposed here and one of the major contributions of this paper.

In the aforementioned works, sparse regression techniques are used, whereby for each state variable $i \in \{1,\dots ,n\}$ the sparse vector $\pmb {\xi }_i$ is estimated as the solution of the following norm-regularized optimization problem:

3 $$\begin{aligned} \pmb {\xi }_i,\mathbf{c }_i \in {{\,\mathrm{arg\,min}\,}}\frac{1}{2m} \sum _{j=0}^{m}||\Theta (\tilde{\mathbf{x }}({\hat{t}}_j)^T,\tilde{\mathbf{u }}({\hat{t}}_j)^T,\mathbf{c })\pmb {\xi }_i - \dot{\tilde{\mathbf{x }}}({\hat{t}}_j) ||_2^2 + \lambda \rho ||\pmb {\xi }_i||_1 + \frac{\lambda (1-\rho )}{2} ||\pmb {\xi }_i||_2^2 \end{aligned}$$

The goal is thus to minimize, in a norm sense, the difference between the value of the state derivatives predicted by the model, $\Theta (\tilde{\mathbf{x }}({\hat{t}}_j)^T,\tilde{\mathbf{u }}({\hat{t}}_j)^T,\mathbf{c })\pmb {\xi }_i$, and the corresponding derivative values $\dot{\tilde{\mathbf{x }}}({\hat{t}}_j)$ that are typically approximated numerically (via e.g. finite difference equations) from the *m* data samples at each sample time ${\hat{t}}_j$. The second part of the expression above is a regularization function composed of the $\ell _1$ and $\ell _2$ norms weighted by parameters $\lambda$ and $\rho$, thereby enforcing the sparsity of the solution by penalizing non-zero values of $\pmb {\xi }$. Note that there is no regularization with respect to parameters $\mathbf{c }$, as $\Xi$ alone determines the sparsity of the recovered governing equations. An $\ell _0$ regularization penalty was considered in^34^ for improved sparsity of the discovered dynamics, which was solved using the SR3 framework^42^ that allows for tackling nonconvex instances of the optimization problem (3).

### Nonlinear dynamic optimization

In contrast to what has been proposed in the literature, in order to *simultaneously* learn the sparse coefficient matrix $\Xi$ and parameters $\mathbf{c }$ in (2), we formulate a constrained dynamic nonlinear program (DNLP) minimizing a given error metric. A discretization scheme using collocation on finite elements^37^ is implemented to convert the candidate model from the continuous-time ODE form in (2) to a system of nonlinear algebraic equations, which is embedded in the constraints of the DNLP^43^. The proposed discretization scheme is A-stable, high-order and can handle nonsmooth events at the element boundaries (e.g., nonsmooth or discontinuous control profiles), an improvement relative to the numerical properties of prior works (e.g.^30,34^), thus making it particularly suitable for stiff differential equations. As an additional contribution, algebraic constraints reflecting lower and upper bounds on the coefficients $\Xi$ and parameters $\mathbf{c }$, derived via pre-processing and/or available domain knowledge, can be seamlessly incorporated in the problem formulation to improve the convergence of the DNLP to the most parsimonious and accurate version of the governing equations (See Methods for further details on how these constraints can be derived). The DNLP is thus formulated in discrete time to minimize the mean $\ell _2$-error plus some regularization term as follows:

4 $$\begin{aligned} \begin{aligned}{}&\min _{\Xi , \mathbf{c },\mathbf{x }}&\frac{1}{2NK} \sum _{i=0}^{N} \sum _{j=0}^{K}||\mathbf{x }(t_{ij})-\tilde{\mathbf{x }}(t_{ij})||_2^2 + \lambda \ell (\Xi )\\&\; \text {s.t.}&\mathbf{g }(\Theta (\mathbf{x }(t_{ij})^T, \tilde{\mathbf{u }}(t_{ij})^T,\mathbf{c}) ,\Xi ) = 0 \\&\;&\Xi \in \{ \Xi ^{L}, \Xi ^{U} \}, \;\;\;\mathbf{c } \in \{ \mathbf{c }^{L}, \mathbf{c }^{U} \} \end{aligned} \end{aligned}$$

where $\mathbf{x }(t_{ij})$ are the states predicted by the candidate governing law at time point $t_{ij}$ (i.e., in finite element *i* and collocation point *j*), the algebraic constrains $\mathbf{g }(\cdot )=0$ represent the discretized version of the nonlinear dynamics given in (2), $\Xi ^{L}, \mathbf{c }^L$ and $\Xi ^{U},\mathbf{c }^U$ are respectively the lower and upper bounds of the estimated coefficients and parameters, and *K* is the number of collocation points on each of the *N* finite elements. The collocation equations form a set of algebraic constraint given by:

5 $$\begin{aligned} \begin{aligned}{}&\left. \frac{d\mathbf{x }(t)}{dt} \right| _{t_{ij}} = \frac{1}{h_i}\sum _{k=0}^{K}\mathbf{x }_{ik}\frac{d\ell _k(\tau _j)}{d\tau }, \;\; j \in \{1,\dots ,K\}, \; i\in \{ 1,\dots ,N\} \\&\left. \frac{d\mathbf{x }(t)}{dt} \right| _{t_{ij}} = \Xi ^T \Theta (\mathbf{x }(t_{ij})^T, \mathbf{u }(t_{ij})^T,\mathbf{c })^T, \;\; j \in \{1,\dots ,K\}, \; i\in \{ 1,\dots ,N\} \\&\mathbf{x }(t_{i+1,0}) = \sum ^{K}_{k=0} \ell _k(1)\mathbf{x }(t_{ik}), \;\; i\in \{1,\dots ,N-1\} \\ \end{aligned} \end{aligned}$$

where $h_i$ is the length of finite element *i*, $t_{i-1} = t_{ij} -\tau _jh_i$, and the state variable $\mathbf{x }(t)$ is interpolated using Lagrange polynomials $\ell _k$ as follows:

6 $$\begin{aligned} \begin{aligned}{}&\mathbf{x }(t) = \sum ^{K}_{k=0}\ell _k(\tau )\mathbf{x }_{ik}, \;\; t\in [t_{i-1}, t_i], \; \tau \in [0,1] \\&\ell _k(\tau ) = \prod ^{K}_{j=0,\ne k} \frac{\tau -\tau _j}{\tau _k-\tau _j} \end{aligned} \end{aligned}$$

The optimal choice of the interpolation points $\tau _j$ is derived in detail in^37^ (Theorem 10.1). Note that the data might need to be resampled (e.g., via interpolation) to estimate the measurements at the defined collocation points. In compact form, we denote (5) and (6) by $\mathbf{g }(\Theta (\mathbf{x }(t_{ij})^T, \tilde{\mathbf{u }}(t_{ij})^T,\mathbf{c}) ,\Xi ) = 0$.

It should be noted that in (4) the basis functions in the dictionary are not directly evaluated on the measurement data $\tilde{\mathbf{x }}$, as is the case in (3). Estimating derivatives directly from data further propagates measurement noise increasing differentiation error, irrespective of the numerical scheme used. Rather, (4) represents a symbolic nonlinear function of the predicted states and does not directly require an approximation of $\tilde{\dot{\mathbf{x }}}$ to derive the coefficients $\Xi$ (as is done in (3)), thus reducing errors related to noise propagation due to numerical differentiation. The proposed approach is analogous to the weak formulations introduced in^27–29^, whereby the system dynamics are expressed in integral from so as not to directly use the estimated derivative in the regression calculation. While the approximation error of (5) is evidently nonzero, as with any discretization scheme, in this case the truncation error is ${\mathcal {O}}(h^{2K-1})$ for Lagrange-Radau collocation points^37^. Thus, the gradient approximation error is expected to be lower than in the case of approaches that directly compute $\dot{\tilde{\mathbf{x }}}$^16,34^, or that use lower order methods^28,30^, as long as a sufficiently high number of finite elements and collocation points is used.

### Moving horizon optimization and thresholding

Evidently, the dimension of the DNLP (4) increases with the granularity of the discretization used, as indicated by *K* and *N*, and indirectly with the dimension the data *m* (i.e., data for longer time horizons require more discretization points for an equivalent approximation error). Similarly, the dimension $n_\theta$ of the dictionary of basis functions (and associated parameterization) is positively correlated with the number of decision variables in (4), as the coefficient matrix has dimensions $\Xi \in {\mathbb {R}}^{n_\theta \times n_x}$. Nonethless, increasing *N*, *K*, *m* or $n_\theta$ are all desirable attributes that improve the likelihood of discovering the true governing equations. Thus, solving (4) while taking into consideration the entire available data set, as is generally done in existing discovery frameworks based on sparse regression^16,25,30,34^, is likely computationally expensive (particularly for granular discretizations and large dictionaries of basis functions). Noting that solving (4) is generally NP-hard, the actual computational effort and solution time cannot be predicted from the above problem dimensions.

An additional fundamental challenge is related to imposing parsimony in the learned model. This sparsification entails eliminating the basis functions that are not part of the true model (1) by setting the corresponding coefficients $\Xi$ to zero. A particular difficulty arises when the true value of a coefficient is “small:” while the corresponding estimate may also be small, it is difficult to discern whether this outcome is correct or the non-zero estimated value is the result of ovefitting (i.e., a spurious attempt to further decrease the value of the objective function in (4) for the training data by increasing model complexity and retaining a larger number of basis functions). A thresholding approach consisting of eliminating terms whose estimated coefficient magnitudes are below a specific value (determined via cross-validation) can in principle be employed^16^, but its performance is expected to degrade with increasing model stiffness and for systems having a broad range of coefficient magnitudes.

The framework proposed here addresses both fundamental challenges described above. To deal with dimensionality, the DNLP in (4) is decomposed into a sequence of lower-dimensional problems defined on shorter time horizons (i.e., using smaller subsets of the available data), for which optimal solutions to (4) can be obtained with significantly lower computational effort. An illustration of the structure of data subsets is shown in Supporting Fig. S.2. After a solution of (4) is computed, a new data subset is selected (intuitively—but not necessarily—by shifting the time window forward by a smaller step than the window size) which is then used to solve (4) again. The repetition of this procedure allows for efficiently learning and refining a sequence of governing equation models each with different coefficient estimates (further details are provided in Algorithm 2 in Methods). This temporal decomposition of the optimization problem is the premise of model predictive control^39^and moving horizon state estimation^38^, by which significant computational speedup has been attained. In conjunction with this moving horizon strategy, the following thresholding claim is made:

#### Claim 1

The coefficients in $\Xi$ corresponding to inactive basis functions typically contribute to overfitting. Small, non-zero values reflect the use of the corresponding functions to fit the noise in the training data. Hence, coefficient estimates derived from the sequence of problems described above that correspond to inactive basis functions are likely to have a relatively high variance.

The converse argument can be made for coefficients of active basis functions: the variance of a sequence of estimates is expected to be relatively low. These claims then support the use of dispersion metrics from statistics (e.g., the coefficient of variation) for the parameters obtained in a sequence of estimates based on subsets of the data, to infer whether a basis function belongs to the true dynamics or not (i.e., if it is active or inactive). In this way, the proposed framework allows for learning dynamics that are truly sparse, similarly to what would be expected from $\ell _0$ regularization for coefficient selection. Once convergence is established (e.g., the structure of the discovered equations does not change after a certain number of iterations), the average or the median of the coefficients can then be used in the aggregated model.

#### Remark 3

The proposed training mechanism is in a way similar to ensemble methods (e.g.,^35,44^) commonly used in machine learning, where a pool of models is trained on different subsets of the data (typically randomly sampled by means of bootstrapping), to reduce variance in the coefficient estimates and thus improve the performance of the final aggregated model prediction.

A comprehensive illustration of our entire proposed framework is shown in Fig. 1 for the Lotka–Volterra predator–prey model, for which the dynamics have the form of $\dot{\mathbf{x }} = \Xi ^T \Theta (\mathbf{x }^T)^T$. That is, the governing equations do not involve control inputs $\mathbf{u }$ or parametric basis functions dependent on $\mathbf{c }$, as in the more general model in (2). We consider this type of systems first in order to demonstrate the performance of our approach and compare it against existing frameworks. Subsequently, we present a case study showing that the proposed framework can cope with dynamics of the form in (2).

### Validation using canonical nonlinear dynamical system examples

**Figure 2:**
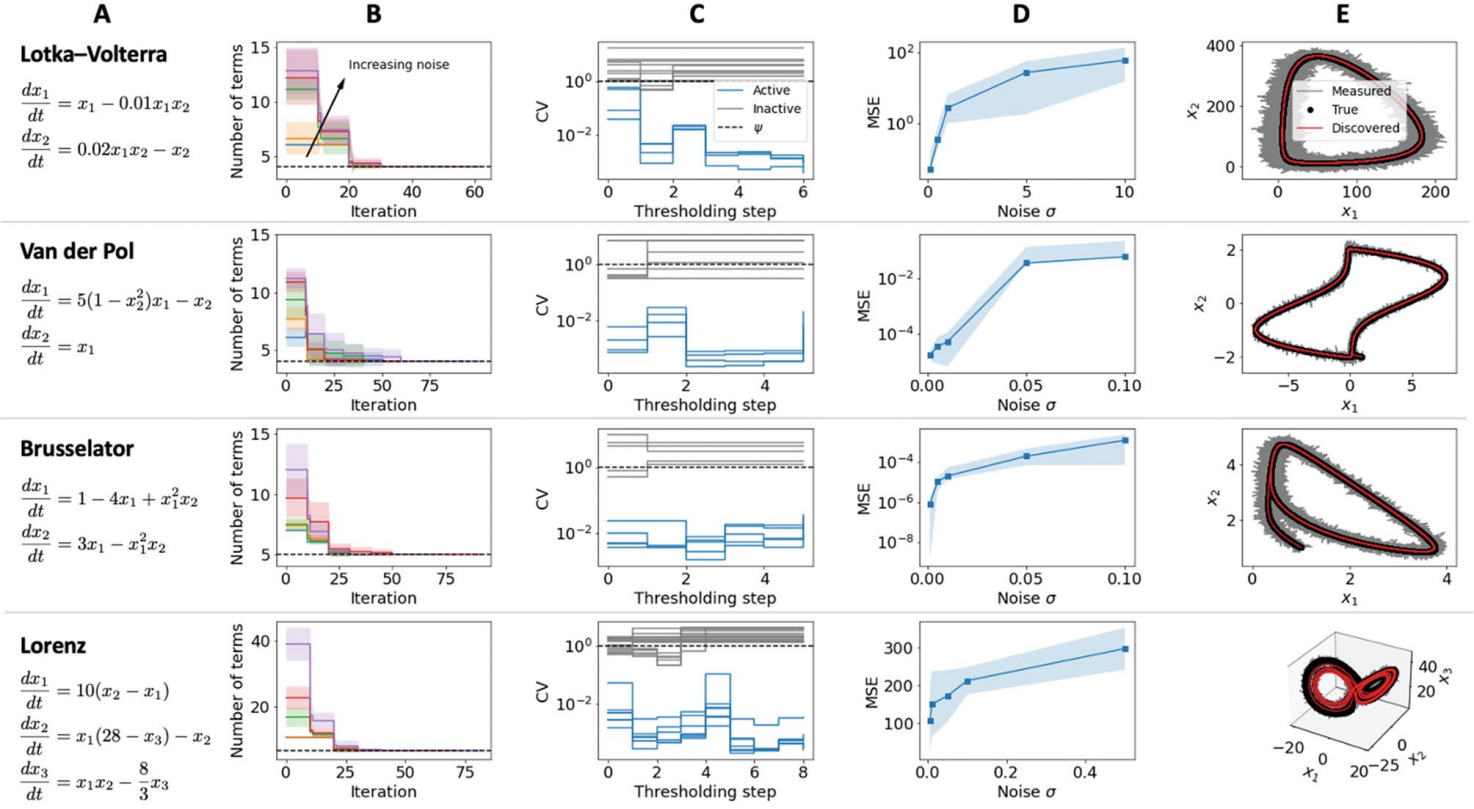
Numerical experiments on canonical nonlinear dynamical systems with governing equations of the form of $\dot{\mathbf{x }} = \Xi ^T \Theta (\mathbf{x }^T)^T$. (**A**) System name and true governing equations. (**B**) Mean number of basis functions in the discovered model as a function of iterations over 10 random samples of the simulated measurement data for each noise level considered (shaded area corresponds to two standard deviations from the mean, dashed black line is used to indicate the number of terms in the true dynamics). Two-dimensional systems were initialized with 28 basis functions, and 3-dimensional systems with 66 (See Supplementary Information for further details). (**C**) Coefficient of variation for each basis function after each thresholding step (results correspond to the highest noise setting considered for each system). (**D**) Average mean squared error (MSE) between simulations of the true dynamics and the discovered dynamics. (**E**) Comparison of discovered dynamics against true model and measured data (results correspond to the highest noise setting considered for each system).

To validate our framework, we consider a series of canonical nonlinear dynamical systems shown in Fig. 2A. For each system, the true model in Fig. 2A was used to generate simulated measurement data which were artificially contaminated with white zero-mean uncorrelated noise of increasing standard deviation $\sigma$. This approach and the amount of noise considered are comparable to prior works in the literature^16,27–29,35^. The results obtained and validation the discovered governing equations are summarized in Fig. 2B–E. The convergence of our method is shown in Fig. 2B, where even for increasing noise levels the number of terms in the discovered equations converges to the black dashed line indicating the number of terms or basis functions in the true dynamics. For data sets with increasing noise variance, the pre-processing step fails to reduce the size of the basis which results in a greater number of iterations to converge to the discovered equations. These results indicate that the proposed moving horizon can cope with larger libraries of basis functions at the expense of a greater number of iterations.

The evolution of the coefficient of variation (CV) of the basis functions retained after pre-processing is shown in Fig. 2C, which is the statistical metric used for the proposed tresholding strategy. The fact that the CV of active variables is noticeably below the established threshold and is considerably lower than for inactive variable, supports and validates the arguments made in Claim 1. Interestingly, per the results in Fig. 2C, active and inactive coefficients can be quantitatively and discretely distinguished which allows for discrete basis function selection, similarly to solving a $\ell _0$ regularized regression problem, while at the same time circumventing the complexities associated with integer programming^26^. We note that for all dynamical systems and noise settings the variability tolerance was set to $\psi = 1$ (a natural choice to distinguish between coefficients with high and low variability, independently of the underlying dynamical systems). Conversely, determining the optimal choice of hyperparameters for prior sparse regression approaches (e.g., determining the regularization penalty for LASSO regression or the coefficient cut-off threshold for sequentially thresholded least squares^16^ is typically highly dependent on and related to the underlying system dynamics and its associated coefficient magnitudes. Further demonstrating the performance of our framework, supporting Figs. S.3 and S.4 show the performance of our approach with different values of hyperparameters including the number of finite elements (*N*) and collocation points (*K*), variability tolerance ($\psi$), and optimization horizon length (*H*). In particular, the results for varying *H* emphasize the scalability advantages associated with the moving horizon scheme employed (i.e., CPU time increases dramatically as *H* approaches the entire time horizon in which the data was sampled). Coarse discretizations lead to high approximation errors, while fine discretizations lead to optimization problems with more variables and constraints, that may be more challenging to solve. Figure S.6 shows the simultaneous effect of measurement noise and sampling frequency on the accuracy and success rate of the proposed discovery framework for the Lotka–Volterra model. The results suggest that the approach maintains good performance even for decreasing sampling frequency (i.e., increasing sparsity of measurement data) when the measurement noise is not substantial.

Figure 2D shows the mean squared error (MSE) between the simulated discovered equations and the true system dynamics for each noise instance considered. While the structural form of the governing equations was correctly identified, increasing measurement noise results in relatively higher error and variance in the values of the estimated coefficients, which intuitively increases the MSE between the two trajectories. Note that the associated coefficients were accurately estimated even for the highest noise instances (the average coefficient estimate error was 0.32%, 1.6%, 0.41%, 2.5% respectively for Lotka–Volterra, van der Pol, Brusselator and Lorenz dynamical systems). Nonetheless, even for the highest noise setting for each system, Fig. 2E indicates that the discovered equations via DySMHO accurately capture the dynamics in the measured data and the overall behavior of the true system dynamics. Similar to ensemble methods^35^, the sequence of coefficient estimates corresponding to iterations where the structure of the discovered equations no longer changes (e.g., convergence) can be used to quantify uncertainty and derive confidence intervals. This characterization then allows for using Monte Carlo sampling strategies to simulate and validate the discovered dynamics for a range of possible coefficient values^45^.

### Benchmarking against existing sparse regression-based discovery frameworks

To evaluate the performance of the present framework relative to state of the art governing equation discovery schemes, we perform benchmarking experiments against several versions of the SINDy algorithm^16^, assessing different available options (mainly the choice of optimization algorithm and the value of the regularization coefficient) using the Python implementation PySINDy^18^. We consider three benchmarks corresponding to conventional SINDy, weak SINDy (W-SINDy)^29^, and ensemble SINDy (E-SINDy)^35^, the latter two being some of the latest developments in the area. All three aforementioned instances of SINDy were tested with three different optimization routines; specifically sequential thresholded least squares (STLSQ)^16^, LASSO regression^14,16^, and sparse relaxed regularized regression (SR3)^34,42^. For the SINDy and E-SINDy methods, that rely on direct state derivative estimation from measurement data, we used the smoothed finite difference option^18^, which employs the Savitzky-Golay filter and was observed to perform the best among the other differentiation methods available. Each combination of SINDy method and optimization solver was screened for a range of hyperparameters for which further details can be found in the Supporting Information. For the present framework, the coefficient of variation thresholding tolerance $\psi$ was the main hyperparmater evaluated, as it dictates the extent of regularization, and for which an intuitive choice for all systems is $\psi = 1$.

**Figure 3:**
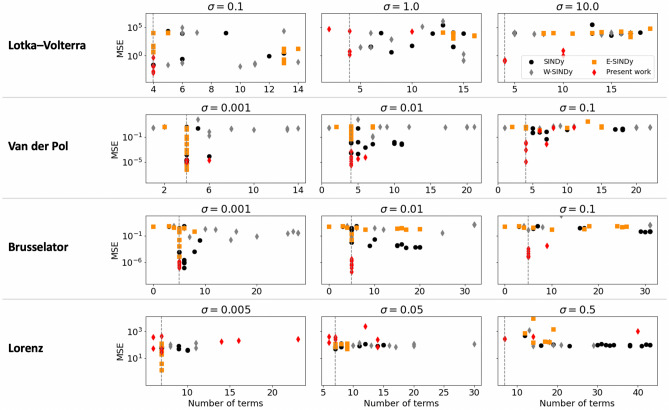
Benchmarking experiments of the proposed framework against the SINDy family of methods. The results shown correspond to the discovered model’s complexity (reflected in the number of terms) versus the model’s predictive ability (reflected in the mean squared error). Each marker corresponds to a specific instance of a given method for a given set of hyperparameters(see Supporting Information for associated values used). The dashed vertical line indicates the number of terms present in the true governing equation for each system.

The benchmarking results for all canonical nonlinear dynamics under increasing noise variance are shown in Fig. 3, where the set of results for each method corresponds to different combinations of hyperparmaters. In order to compute the complexity for SINDy models, we set all “small” (but nonzero) coefficients estimates to zero if their absolute values were lower than 10% of the smallest coefficient in the true governing equations. While this alteration favors the complexity score of the models identified with SINDy (i.e., it yields a lower complexity than was actually identified), it highlights the inherent advantage of our proposed thresholding method which results in truly sparse dynamics (i.e. the coefficients of basis functions that are deemed inactive are set exactly to zero). This property, together with the more intuitive selection of the thresholding tolerance $\psi$ for the present work, results in discovering models that are generally sparser than those identified via SINDy methods, as can be noted from the results shown in Fig. 3. We note that, in particular, SINDy methods failed to eliminate trigonometric basis functions ($\sin (\mathbf{x })$ and $\cos (\mathbf{x })$) and constant terms, which are likely low in magnitude relative to the measurement data and serve to reduce MSE by overfitting noise. The coefficients associated with these (inactive) basis functions exhibit considerable variability when estimated on sequential data sets, and thus are pruned in our framework, resulting in identified governing equations of relatively lower complexity.

For low noise instances, our approach exhibits comparable performance (in terms of MSE and model complexity) to that of the different SINDy methods when the appropriate regularization parameter is used. We note that for the Lorenz oscillator dynamics, due to its chaotic nature, even very minor coefficient estimation errors can result in significant deviations between the simulated trajectories and the original model, which in turn results in a large MSE for the discovered model. For example, for the present framework, the average coefficient error was 2.23% with standard deviation of 0.14 for the highest noise instance corresponding to $\sigma =0.5$. The relative performance of our proposed approach clearly improves for increasing measurement noise across all dynamical system examples. While in some instances the proposed framework fails to discover the correct model for large and small values of hyperparameter $\psi$, it consistently identifies the true dynamics for $\psi \approx 1$, which is an intuitive setting to distinguish between high and low variance coefficients (as was empirically demonstrated in Fig. 2C). Further, the high-order implicit dicretization in the proposed framework appears to be advantageous for dynamical systems where the order of magnitude of the coefficients is very different (e.g., Lotka Volterra), and for systems with stiff dynamics (e.g., van der Pol and Brusselator).

### Validation using a non-isothermal chemical reactor system with exogenous inputs

To further demonstrate the advantages of the proposed framework relative to the previously considered sparse regression frameworks^16,18,29,35^, we consider a case study corresponding to a controlled non-isothermal continuously stirred tank reactor (CSTR) system. A similar non-isothermal case study was reported previously in^45^, where further details regarding the system can be found. We note that SINDy methods have been previously applied for inferring chemical kinetics^22,24^ but, unlike the current case, only isothermal reaction conditions were considered. In particular, we show that our moving horizon nonlinear optimization scheme can recover governing equations that are of the form of $\Xi ^T\Theta (\mathbf{x }(t)^T,\mathbf{u }(t)^T, \mathbf{c })^T$, with exogenous inputs $\mathbf{u }(t) \in {\mathbb {R}}^{n_u}$ (which can be used as an input signal to excite the dynamics or to drive the states of the system to a desired target value (setpoint)) and parametric basis functions. The governing dynamics of the CSTR are given by the coupled material and energy balance equations:

7 $$\begin{aligned} \begin{aligned}{}&\frac{dC_A(t)}{dt} = \frac{q}{V}(C_{A,i} - C_A(t))-k_0 e^{-E_a/RT(t)}C_A(t) \\&\frac{dT(t)}{dt} = \frac{q}{V}(T_i -T(t)) + \frac{(-\Delta H_R)}{\rho C}k_0 e^{-E_a/RT(t)}C_A(t)+\frac{U{\mathscr {A}}}{V \rho C}(T_c(t)-T(t)) \end{aligned} \end{aligned}$$

where $C_A(t)$ and *T*(*t*) are the system states $\mathbf{x }(t)$ and represent, respectively, the concentration of species *A* and the temperature. Further, $\rho$ is the density of the liquid, *C* is its heat capacity, $(\Delta H_R)$ is the heat of reaction, $T_i$ is the temperature of the inlet stream, $T_c$ is the temperature of the coolant, *U* is the overall heat transfer coefficient, ${\mathcal {A}}$ is the heat transfer area, $k_0$ is the pre-exponential factor, $E_a$ is the activation energy, and *R* is the universal gas constant. Differently from^45^, that considers *q*(*t*) as the manipulated input in the isothermal case, this example assumes $T_c(t)$ to be the manipulated input in a non-isothermal setting in which the temperature dynamics in (7) must be accounted for. Further details regarding the case study parameters and hyperparameters used can be found in the Supporting Information.

**Figure 4:**
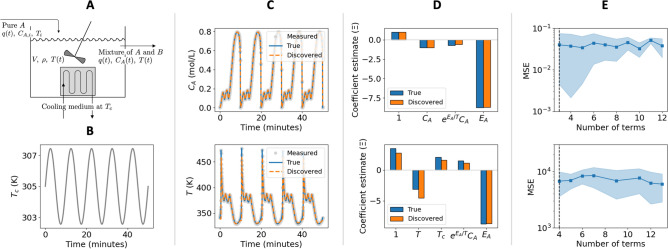
Numerical experiments on CSTR case study with dynamics of the form of $\dot{\mathbf{x }} = \Xi ^T \Theta (\mathbf{x }^T, \mathbf{u }^T,\mathbf{c })^T$. (**A**) Schematic of CSTR system showing states, inputs, and key parameters. (**B**) Control input signal used to excite dynamics given by $T_c(t)=305\times (1+\sin (\pi t/5)/125)$. (**C**) State variable trajectories (composition (top) and temperature (bottom)) corresponding to: measurements with noise, true trajectory, and sample discovered trajectory. (**D**) Comparison of coefficient values of the true governing equation and that of the discovered dynamics (coefficient estimates were scaled to be of the same order of magnitude). (**E**) Average MSE as a function of the number of terms remaining in the dynamics throughout the iterations for ten randomly generated data sets (shaded area corresponds to two standard deviations from the mean). Dashed vertical line indicates the number of basis functions present in the true governing equations.

Results for the discovery of the CSTR governing mass and energy balances are shown in Fig. 4, corresponding to ten randomly generated data instances for which the true functional forms of the equations in (7) were identified. The discovered equations correctly capture the dynamics for both composition and temperature as shown in Fig. 4C, accurately reproducing the slow and fast frequency of the oscillations observed in the data. Nonetheless, similarly to chaotic systems (e.g., Lorenz oscillator in Fig. 2), it should be the noted that for stiff and multi-scale systems such as this CSTR example, even small parameter estimation errors as the ones observed in Fig. 4D can result in significant deviations from the true simulated trajectory. The plots in Fig. 4E show that as the algorithm proceeds and an increasing number of basis functions is pruned from the dictionary, the predictive capability (in terms of MSE) of the discovered model remains relatively constant, which is indicative of convergence to the most parsimonious governing equation.

## Discussion

### Limitations and directions of future work

While the numerical experiments performed show that our proposed framework has multiple promising features and benefits relative to existing approaches, there are several future research questions to be addressed. For example, the open source code developed for the present framework is only capable of handling ODEs and in its current from cannot cope with systems governed by PDEs. Nonetheless, we note that the orthogonal collocation equations introduced in (5) and (6) readily generalize to differential operators other than time derivatives (e.g., spatial and higher order derivatives), such that the proposed scheme can be used to identify sparse PDEs. We believe that the implicit integration scheme embedded in our proposed method would be advantageous for high-order PDE systems, for which estimation of high-order derivatives from noisy measurement data is especially challenging^29^. Developing the associated code and performing validating experiments is a crucial next step.

An interesting potential extension to the present work is an adaptive thresholding tolerance $\psi$, whose value decreases with increasing number of iterations and basis functions pruned. This idea stems from the fact that, as fewer functions remain in the basis, the variability of the remaining inactive coefficients decreases making the thresholding step more challenging. This property can be observed from the results presented in Fig. 2B, where the first few thresholding steps eliminate a considerably larger number of basis functions than the later steps before convergence is established. For high noise environments, even the coefficient estimates of active basis functions are likely to have high variability and could be erroneously pruned if $\psi$ is initially set to a low value when the library of basis functions is large.

Furthermore, for higher dimensional systems it is important to employ efficient large-scale nonlinear programming strategies. Developing numerical solutions that exploit the (sparse) structure of the collocation equations and resulting optimization problem is a key step in discovering governing equations for systems of industrially-relevant dimensions^46^. In particular, decomposition and parallelization schemes are critical for considering larger data sets, while maintaining manageable computational complexity^37^. While for the present work local optimization algorithms were employed, to solve (4), we expect that using scalable global optimization solvers could further improve the accuracy of the model coefficient estimates, and the overall performance of the proposed framework.

### Conclusions

Data-driven discovery of governing equations is a promising avenue for advancing our understanding of and elucidating new phenomena across a wide range of disciplines. This new fundamental knowledge can in turn be used to drive the development of new technologies to solve pressing scientific and societal challenges. In this paper, we introduced and validated a novel moving horizon-based, nonlinear dynamic optimization framework for learning governing equations from noise-contaminated state measurements over time. The proposed framework benefits from the properties of weak (or integral) discovery methods by incorporating high order and stable numerical integration schemes to represent the system dynamics, thereby minimizing gradient approximation errors, and improving performance for stiff and multiscale nonlinear systems. The proposed moving horizon scheme not only improves computational tractability of the underlying optimization problem, but provides a systematic and statistically meaningful approach for thresholding basis functions by which the identified equations are generally of lower complexity than those resulting from sparse regression approaches proposed by other authors. The sequential coefficient estimates are aggregated once the structure of the discovered equations does not change, improving robustness to noisy measurements in a similar sense to previously reported ensemble approaches. Lastly, the nonlinear programming structure that lies at the core of the proposed methodology is capable of handling more complex dynamical systems by relaxing the assumption made in most prior works (i.e., that the dynamics are linear with respect to the unknown model parameters), as it was demonstrated empirically for a chemical reactor case study.

## Methods

### Pre-processing: data smoothing

The Savitzky-Golay filter (SVGF) in the SciPy package (https://www.scipy.org/) was employed, which is based on local least-squares regression polynomial approximations applied to the data on moving windows of a given size. An iterative scheme was developed to automatically determine the appropriate smoothing widow size, which consisted of smoothing the measured signal using increasing window size until the estimated noise component did not change significantly (given a specific tolerance). Further details are presented in Algorithm 1, and an illustration is shown in the Supplementary Fig. S.1.
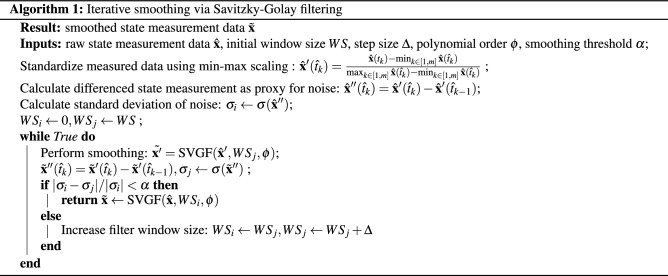


#### Pre-processing: statistical analysis

The smoothed state measurements are arranged in the form of a data matrix $\tilde{\mathbf{X }}$:

8 $$\begin{aligned} \tilde{\mathbf{X }} =\begin{bmatrix} \tilde{\mathbf{x }}^{T}({\hat{t}}_1) \\ \vdots \\ \hat{\mathbf{x }}^{T}({\hat{t}}_m) \end{bmatrix} = \begin{bmatrix} {\tilde{x}}_1 ({\hat{t}}_1) &{} \dots &{} {\hat{x}}_n ({\hat{t}}_1) \\ \vdots &{} \ddots &{} \vdots \\ {\tilde{x}}_1 ({\hat{t}}_m) &{} \dots &{} {\hat{x}}_n ({\hat{t}}_m) \\ \end{bmatrix} \end{aligned}$$

where $t_1$, $t_2$, $\dots$, $t_m$ are the time intervals at which the measurements were collected. The dictionary of candidate basis functions is then evaluated for every point in the data matrix, resulting in a structure such as the one below:9where $\tilde{\mathbf{X }}^{P_2}$ denotes second order polynomials which may include interaction terms, e.g.:

10 $$\begin{aligned} \tilde{\mathbf{X }}^{P_2} = \begin{bmatrix} \tilde{\mathbf{x }}^{P_2}({\hat{t}}_1) \\ \vdots \\ \tilde{\mathbf{x }}^{P_2}({\hat{t}}_m) \end{bmatrix} = \begin{bmatrix} {\tilde{x}}_1^2({\hat{t}}_1) &{} {\tilde{x}}_1({\hat{t}}_1) {\tilde{x}}_2({\hat{t}}_1) &{} \cdots &{} {\tilde{x}}_2^2({\hat{t}}_1) &{} \cdots &{} {\tilde{x}}_n^2({\hat{t}}_1)\\ {\tilde{x}}_1^2({\hat{t}}_2) &{} {\tilde{x}}_1({\hat{t}}_2) {\tilde{x}}_2({\hat{t}}_2) &{} \cdots &{} {\tilde{x}}_2^2({\hat{t}}_2) &{} \cdots &{} {\tilde{x}}_n^2({\hat{t}}_2) \\ \vdots &{} \vdots &{} \ddots &{} \vdots &{} \ddots &{} \vdots \\ {\tilde{x}}_1^2({\hat{t}}_m) &{} {\tilde{x}}_1({\hat{t}}_m) {\tilde{x}}_2({\hat{t}}_m) &{} \cdots &{} {\tilde{x}}_2^2({\hat{t}}_m) &{} \cdots &{} {\tilde{x}}_n^2({\hat{t}}_m) \end{bmatrix} \end{aligned}$$

Evidently, the form of this structure will depend on the choice of basis functions. Using this form, Granger causality tests (based on F- and chi-squared distributions) were used to determine potential causality between $\Theta _i (\tilde{\mathbf{X }}_j({\hat{t}}_{k-1}))$ and $\tilde{\mathbf{X }}_j({\hat{t}}_k)$ for all basis functions $i\in \{1,\dots ,n_\theta \}$ and states $j\in \{1,\dots ,n_x\}$. Basis functions *i* for which the null hypothesis (that $\Theta _i(\tilde{\mathbf{X }}_j)$ does not Granger-cause ${\tilde{\mathbf{X }}}_j$) could not be rejected with high significance, were eliminated from the library. Stationarity of the time series was checked using the Dickey-Fuller test and enforced by differencing as needed. The statsmodel (https://www.statsmodels.org/stable/index.html) implementations of the Granger causality and Dickey-Fuller tests were used.

Next, the derivative of each state variable is approximated from the data by using central finite differences as follows:

11 $$\begin{aligned} \dot{\tilde{\mathbf{x }}}({\hat{t}}_k) = \frac{\tilde{\mathbf{x }}({{\hat{t}}_{k+1}})-\tilde{\mathbf{x }}({{\hat{t}}_{k-1}})}{{\hat{t}}_{k+1}-{\hat{t}}_{k-1}}, \;\; k = 2,\dots ,m-1 \end{aligned}$$

and similar to (9) a data derivative matrix $\dot{\tilde{\mathbf{X }}}$ is formed. Other derivative approximation strategies (e.g.,^47^) can be used when there is significant noise in the data. Ordinary least squares (OLS) regression was used to obtain initial coefficient estimates for $\Xi$ in (4) as well as the associated upper and lower bounds ($\Xi ^L$ and $\Xi ^U$). The linear system solved is given by:

12 $$\begin{aligned} \dot{\tilde{\mathbf{X }}} = \Theta (\tilde{\mathbf{X }})\Xi ^{OLS} \end{aligned}$$

This allows for performing preliminary variable selection by computing the F-statistic and p-value associated with each coefficient estimate, as well as for using the resulting confidence intervals (for a specified confidence level) to serve as ($\Xi ^L$ and $\Xi ^U$) in (9). The statsmodel implementation of OLS was used to solve the regression problem (12), as well as to compute associated test statistics and confidence intervals.

### Discretization of the candidate ordinary differential equations

The collocation equations in (5) and (6) are represented in compact form as $\mathbf{g }(\Theta (\mathbf{x }(t_{ij})^T, \mathbf{u }(t_{ij})^T),\mathbf{c }),\Xi )=0$ in the DNLP in (4). We employ the pyomo.DAE (http://www.pyomo.org/) modeling extension that enables automatic simultaneous discretization of ODEs, and leverages Gauss-Legendre and Gauss-Radau collocation schemes to determine the interpolating points in (5) and (6). It should be noted that the optimal choice of finite elements and collocation points may not align with the sample times ${\hat{t}}_1, \dots , {\hat{t}}_m$ at which the data were originally collected, thus spline interpolation is required to approximate the data at the relevant time instants. We employ the SciPy package in Python using a cubic spline to estimate the state values at the collocation points.

### Moving horizon optimization approach

A detailed outline of the moving horizon algorithm is presented next in Algorithm 2 and an illustration is shown in Supplementary Fig. S.2. In brief, the algorithm consists of solving (4) and performing parameter thresholding every $\omega$ iterations (the thresholding process is described separately in Algorithm 3). The algorithm terminates when the training data are exhausted or when convergence is established, that is when the number of basis functions remaining in the library, denoted as $|\Theta |$, does not change after a number $\Omega$ of thresholding steps.
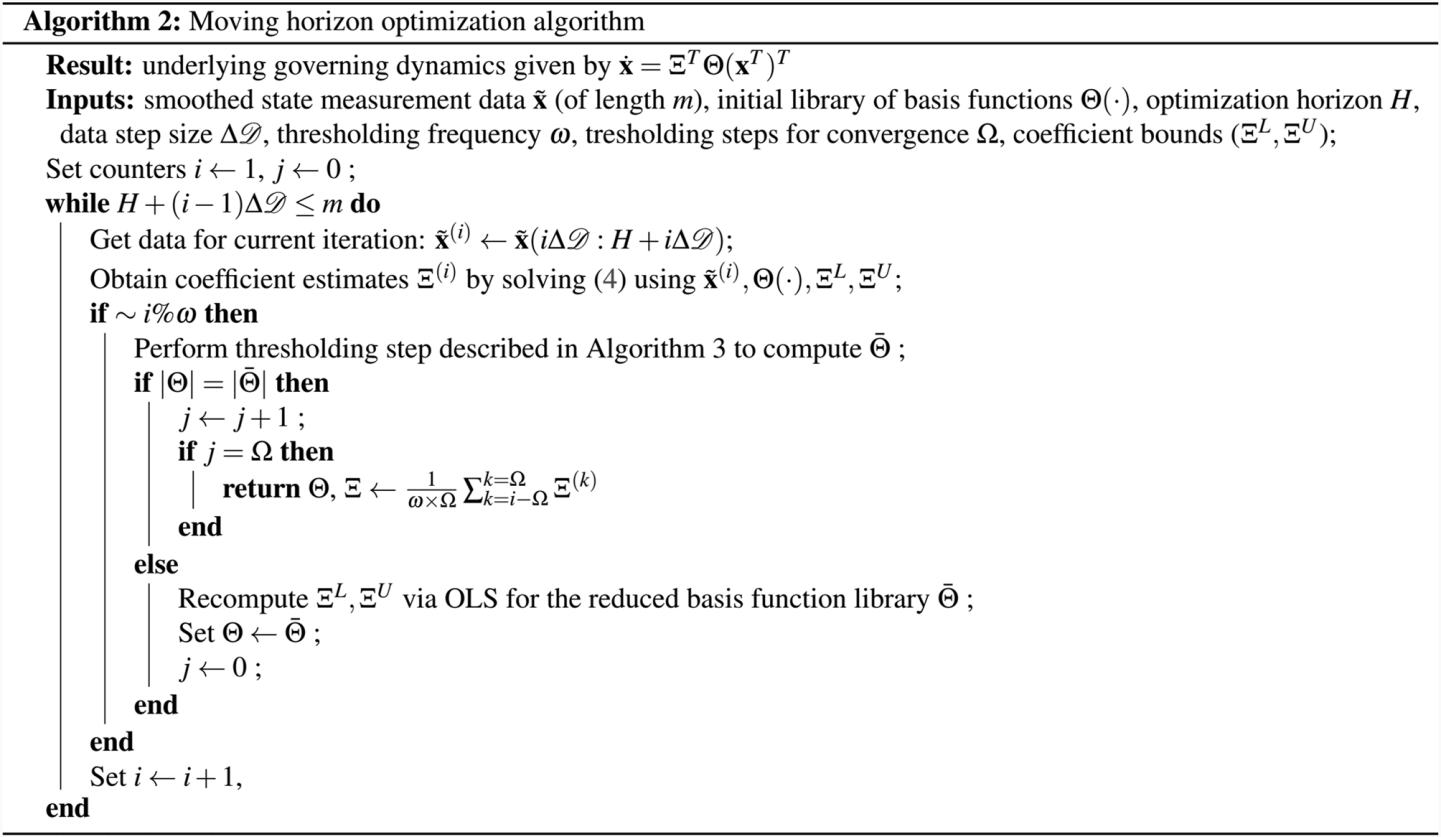


Evidently, the number of optimization problems to be solved depends directly on the choice of optimization horizon *H*. Nevertheless, the problems in the sequence are likely significantly more computationally tractable than solving (4) for the entire (large-scale) data set. It is worth mentioning that, to date, no analytical frameworks exist for determining the optimal choice of horizon *H*. The empirical consensus is that longer horizons yield better results (i.e., convergence of the estimates to the true values of the parameters), which intuitively comes at a computational cost^38^. For periodic systems, such as the Lotka–Volterra system shown in Supplementary Fig. S.2, an intuitive choice for *H* can be an integer multiple of the period corresponding to the fundamental oscillation frequency, which can be estimated from the data.

#### Thresholding algorithm

The proposed thresholding approach is described in Algorithm 3, and is embedded within the moving horizon scheme introduced previously in Algorithm 2. The variability of the sequence of coefficient estimates can be quantified statistically by computing the coefficient of variation (*CV*), defined as the ratio between the standard deviation and the mean. In our framework, the coefficient of variation is computed for the coefficients of all basis functions $\Theta _\theta \; \forall \theta \in \{1,\dots ,|\Theta |\}$, and for all state variables $j\in \{1,\dots ,n_x\}$. If the coefficient of variation $CV_{\theta ,j}$ is greater than the specified variability threshold $\psi$, then the basis function is pruned and not considered in future iterations of the moving horizon scheme. Otherwise, the basis function $\Theta _\theta$ remains in the dictionary.
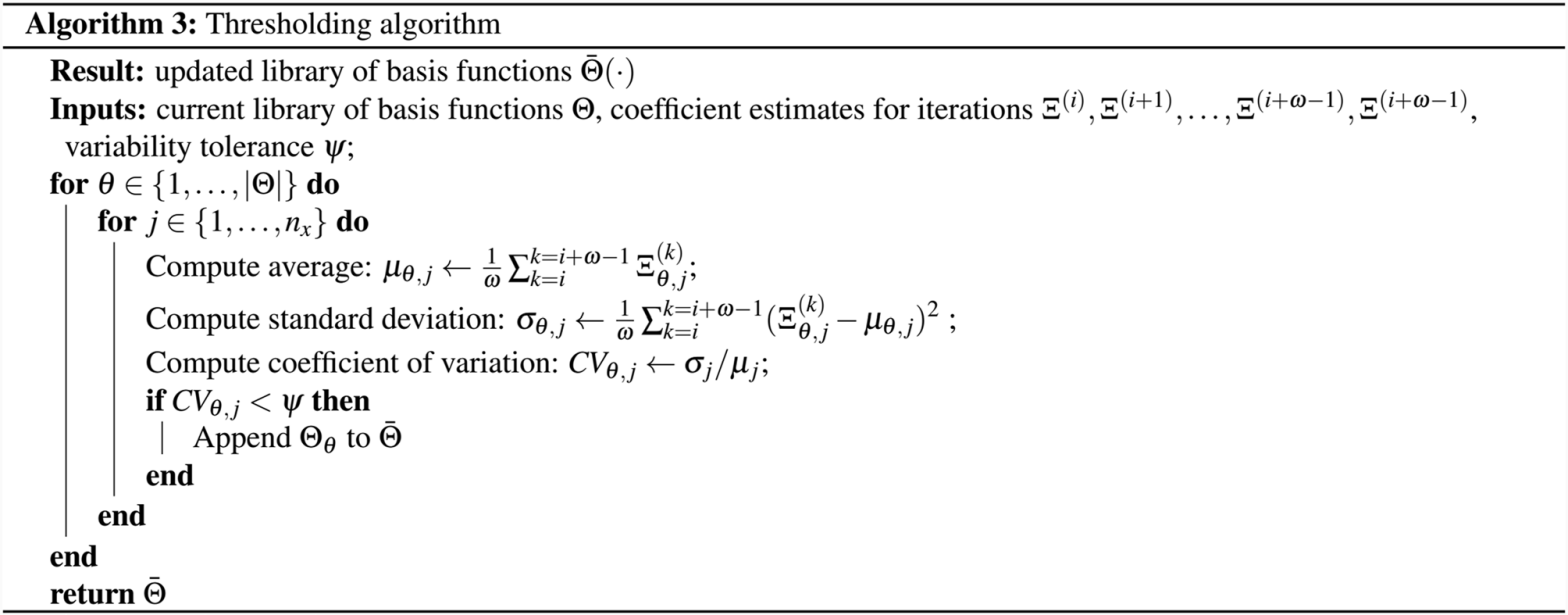


Basis functions such as $\{\pmb {1}, \mathbf{x }\} \in \Theta$ are particularly prone to contributing to overfitting noise components in the data, as well as to the differentiation error introduced when the dynamics are discretized (particularly in high noise environments and when the initial function library is larger). To prevent spurious thresholding for this type of basis functions, whose associated coefficients are likely so see greater variability across different data subsets, it is preferable to retain them in the basis for the first few thresholding steps regardless of their associated coefficients’ observed *CV*. After these initial iterations and when (potentially) some of the other inactive basis functions have been pruned, if basis functions like e.g. $\{\pmb {1}, \mathbf{x } \}$ are in fact active they are expected to experience less variability in their respective coefficients and remain in the basis when the algorithm converges.

## Supplementary Information

Supplementary Information.

## Data Availability

All data and code used in this analysis can be found at: https://github.com/Baldea-Group/DySMHO.
