# Supplementary material for: Data-driven discovery of the governing equations of dynamical systems via moving horizon optimization
Source: Sci Rep. 2022 Jul 12;12:11836. doi: 10.1038/s41598-022-13644-w (PMC9276674; doi:10.1038/s41598-022-13644-w)
Supplement: Supplementary file 1 — Supplementary Information. [file 41598_2022_13644_MOESM1_ESM.pdf]

Supplementary Information: Data-driven discovery of the governing equations of dynamical systems via moving horizon optimization

Fernando Lejarza,[1] Michael Baldea,[1,2,*]

[1] The University of Texas at Austin, McKetta Department of Chemical Engineering
[2] The University of Texas at Austin, Oden Institute for Computational Engineering and Sciences
*To whom correspondence should be addressed; E-mail: `mbaldea@utexas.che.edu`

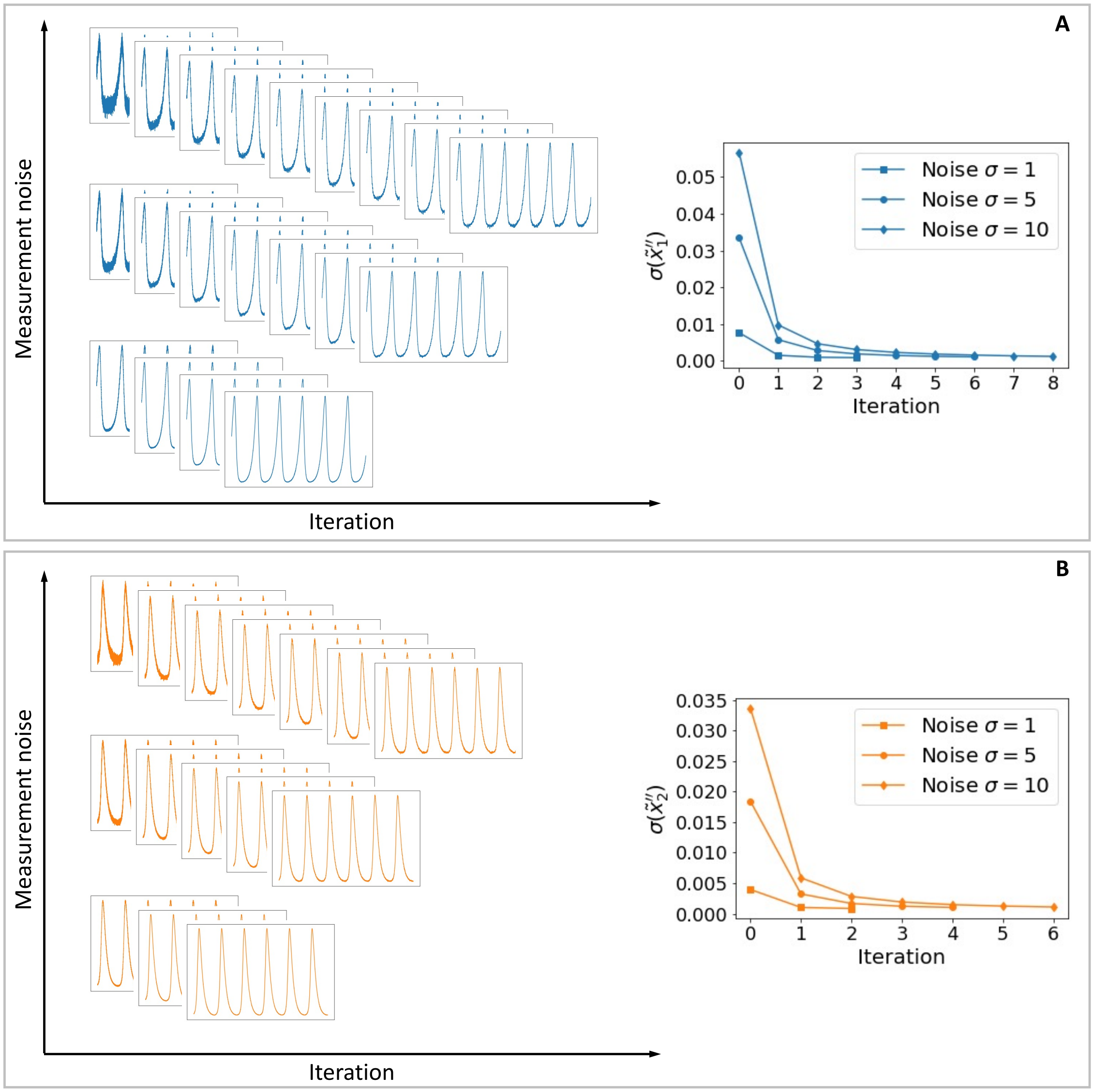

Figure S.1: Illustration of smoothing algorithm for Lotka-Volterra predator-prey model. (**A**) results obtained for state x_1. (**B**) results for state x_2. Figures on the left show the smoothed system trajectories as a function of initial measurement noise at every iteration of the smoothing algorithm, figures on the right show the standard deviation of the estimated noise at every iteration. (Results obtained using $\alpha = 0.1$, $WS_i = 10$, $\Delta = 10$, and $\phi = 2$)

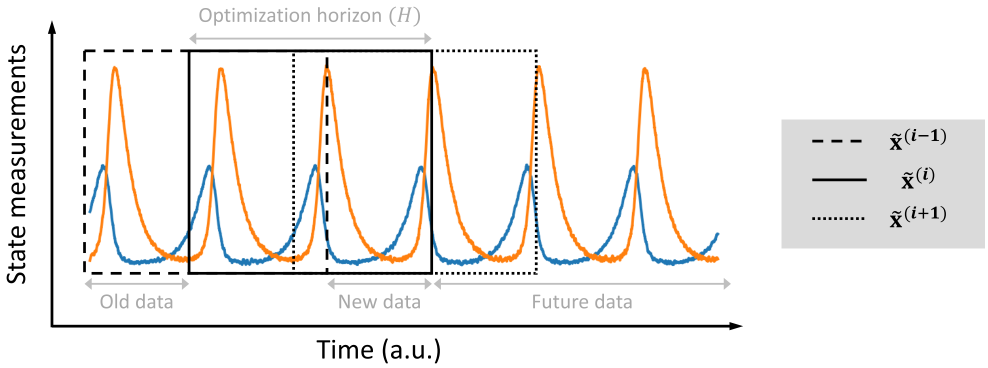

Figure S.2: Illustration of subsets of data of length H used for the proposed moving horizon algorithm using simulated and smoothed state measurements corresponding to the Lotka-Volterra predator-prey model. Data frames used for the past iteration, current iteration, and next iteration are denoted as $\tilde{\mathbf{x}}^{(i-1)}$, $\tilde{\mathbf{x}}^{(i)}$, and $\tilde{\mathbf{x}}^{(i+1)}$, respectively.

Configurations for numerical experiments

All computations were performed on a PC running Windows 7 64-bit, with a 3.6 GHz Intel Core i7-7700 processor and 32 GB RAM. All DNLPs where formulated in Python 3.8.3 using `Pyomo` [1], and solved using CONOPT [2] (a feasible path solver based on the Generalized Reduced Gradient (GRG) nonlinear solution method) as the nonlinear solver using all default settings. The objective function minimized was the mean squared difference between the model and the state measurements, without regularization (i.e., $\lambda = 0$).

Canonical nonlinear dynamical systems

The basis functions for the Lotka-Volterra, van der Pol, and Brusselator examples were:

$$\begin{aligned} \Theta_{x_1} &= \{1, x_1, x_2, x_1x_2, x_1^2, x_2^2, x_1^2x_2, x_1x_2^2, x_1^3, x_1^4, 1/x_1, e^{x_1}, \sin x_1, \cos x_1\} \\ \Theta_{x_2} &= \{1, x_2, x_1, x_1x_2, x_2^2, x_1^2, x_2^2x_1, x_2x_1^2, x_2^3, x_2^4, 1/x_2, e^{x_2}, \sin x_2, \cos x_2\} \end{aligned} \tag{S.1}$$

and for the Lorenz example were:

$$\begin{aligned} \Theta_{x_1} = &\{1, x_1, x_2, x_3, x_1x_2, x_1x_3, x_2x_3, x_1^2, x_2^2, x_3^2, x_1^2x_2, x_1x_2^2, x_1^2x_3, x_1x_3^2, x_2^2x_3, \\ &x_2x_3^2, x_1^3, x_1^4, 1/x_1, e^{x_1}, \sin x_1, \cos x_1\} \\ \Theta_{x_2} = &\{1, x_2, x_1, x_3, x_2x_1, x_2x_3, x_1x_3, x_2^2, x_1^2, x_3^2, x_2^2x_1, x_2x_1^2, x_2^2x_3, x_2x_3^2, x_1^2x_3, \\ &x_1x_3^2, x_2^3, x_2^4, 1/x_2, e^{x_2}, \sin x_3, \cos x_3\} \\ \Theta_{x_3} = &\{1, x_3, x_1, x_2, x_3x_1, x_3x_2, x_1x_2, x_3^2, x_1^2, x_2^2, x_3^2x_1, x_3x_1^2, x_3^2x_2, x_3x_2^2, x_1^2x_2, \\ &x_1x_2^2, x_3^3, x_3^4, 1/x_3, e^{x_3}, \sin x_3, \cos x_3\} \end{aligned} \tag{S.2}$$

Tables S.1-S.2 outline all of the parameters and configurations used for the numerical experiments on canonical nonlinear dynamical systems.

Table S.1: Configuration for Lotka-Volterra and van der Pol oscillator numerical experiments

**System**	**Configuration**	
Lotka-Volterra	Data simulation	Initial conditions: (100,15)
		Sampling frequency: 1/500
	Smoothing	$WS = 10$
		$\gamma = 10$
		$\alpha = 0.1$
	Pre-processing	Granger tests: χ^2 and F-distributions
		Granger p-value: 0.1
		OLS p-value: 0.9
		OLS % confidence: 1×10^{-6}
	Discretization	Scheme: Lagrange-Radau
		Finite elements: 50
		Collocation points: 15
		Data interpolation: Cubic spline
	Moving horizon	H: 6 (3,000 data samples)
		$\Delta\mathcal{D}$: 100 samples
		ω: 10
		Ω: 40
	Thresholding	ψ: 1
Van der Pol	Data simulation	Initial conditions: (1,-2)
		Sampling frequency: 1/500
	Smoothing	$WS = 10$
		$\gamma = 10$
		$\alpha = 0.1$
	Pre-processing	Granger tests: χ^2 and F-distributions
		Granger p-value: 0.1
		OLS p-value: 0.8
		OLS % confidence: 1×10^{-6}
	Discretization	Scheme: Lagrange-Radau
		Finite elements: 80
		Collocation points: 15
		Data interpolation: Cubic spline
	Moving horizon	H: 20 (40,000 data samples)
		$\Delta\mathcal{D}$: 50 samples
		ω: 10
		Ω: 40
	Thresholding	ψ: 1

Table S.2: Configuration for Brusselator and Lorenz oscillator numerical experiments

**System**	**Configuration**	
Brusselator	Data simulation	Initial conditions: (1,1)
		Sampling frequency: 1/1000
	Smoothing	$WS = 10$
		$\gamma = 10$
		$\alpha = 0.1$
	Pre-processing	Granger tests: χ^2 and F-distributions
		Granger p-value: 0.1
		OLS p-value: 0.8
		OLS % confidence: 1×10^{-6}
	Discretization	Scheme: Lagrange-Radau
		Finite elements: 60
		Collocation points: 15
		Data interpolation: Cubic spline
	Moving horizon	H: 10 (10,000 data samples)
		$\Delta\mathcal{D}$: 100 samples
		ω: 10
		Ω: 40
	Thresholding	ψ: 1
Lorenz	Data simulation	Initial conditions: (-8,8,27)
		Sampling frequency: 1/1000
	Smoothing	$WS = 10$
		$\gamma = 10$
		$\alpha = 0.1$
	Pre-processing	Granger tests: χ^2 and F-distributions
		Granger p-value: 0.1
		OLS p-value: 0.7
		OLS % confidence: 1×10^{-6}
	Discretization	Scheme: Lagrange-Radau
		Finite elements: 50
		Collocation points: 15
		Data interpolation: Cubic spline
	Moving horizon	H: 2 (2,000 data samples)
		$\Delta\mathcal{D}$: 100 samples
		ω: 10
		Ω: 40
	Thresholding	ψ: 1

Hyperparameter sensitivity experiments for Lotka-Volterra system

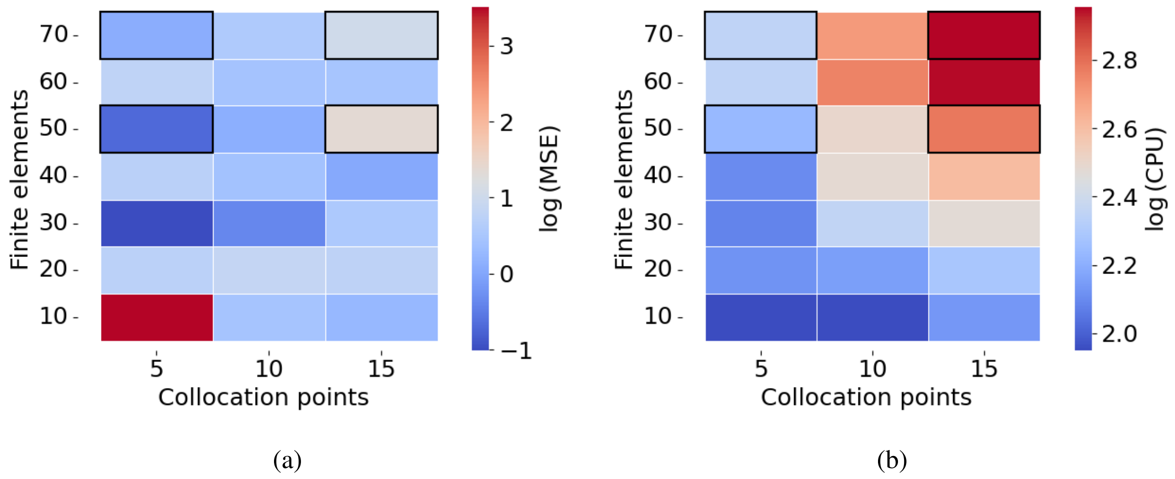

Figure S.3: Sensitivity of our framework to the number of finite elements and collocation points used to discretize the underlying governing equation (all other configurations as in Table S.1 held constant). (a) Shows the resulting mean squared error, and (b) shows the resulting CPU time in seconds. Black edges highlight instances in which the algorithm did not arrive to the exact structural form of the dynamics.

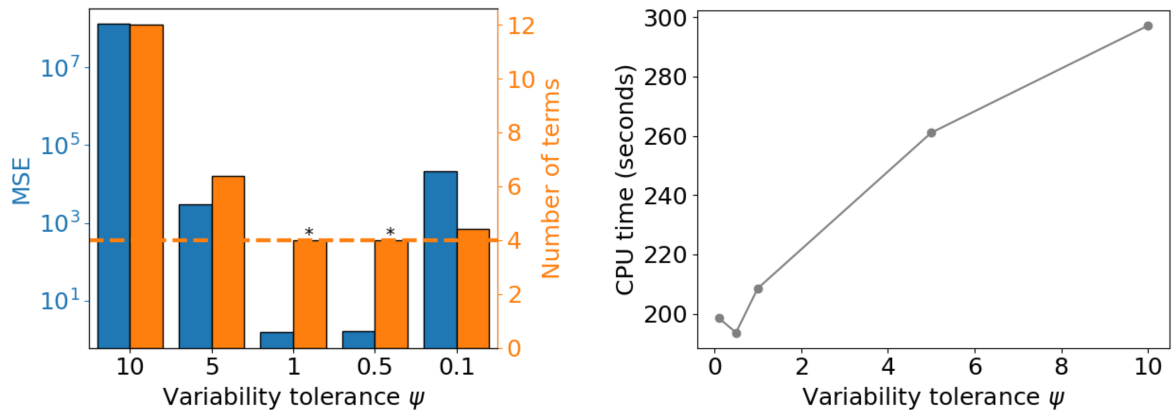

(a)

(b)

Figure S.4: Results for Lotka-Volterra system as a function of different coefficient of variation thresholds ψ (all other configurations as in Table S.1 held constant). Results shown correspond to the average of five randomly sampled simulated measurement data using $\sigma = 5$. (a) Average mean squared error (MSE) and average number of discovered terms (horizontal dashed line indicates the number of terms in the true governing equations, and asterisk are used to denote ψ values in which the correct dynamics were discovered for all five instances). (b) Average CPU time required to solve moving horizon dynamic optimization problem.

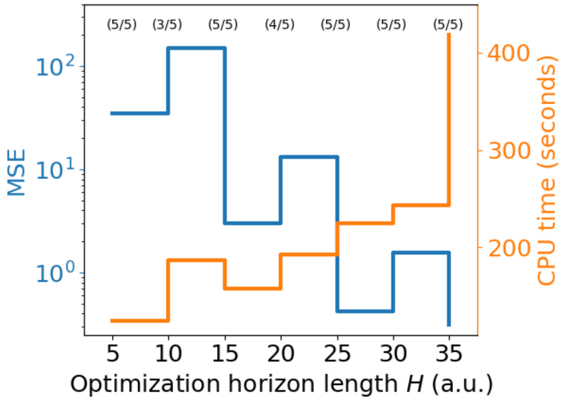

Figure S.5: Results for Lotka-Volterra system as a function of different optimization horizon lengths H (all other configurations as in Table S.1 held constant). Results shown correspond to the average of five randomly sampled simulated measurement data using $\sigma = 5$. Trajectories are shown for the average MSE and CPU time, and annotations on top denote the number of instances for which the true structure of the dynamics was correctly discovered.

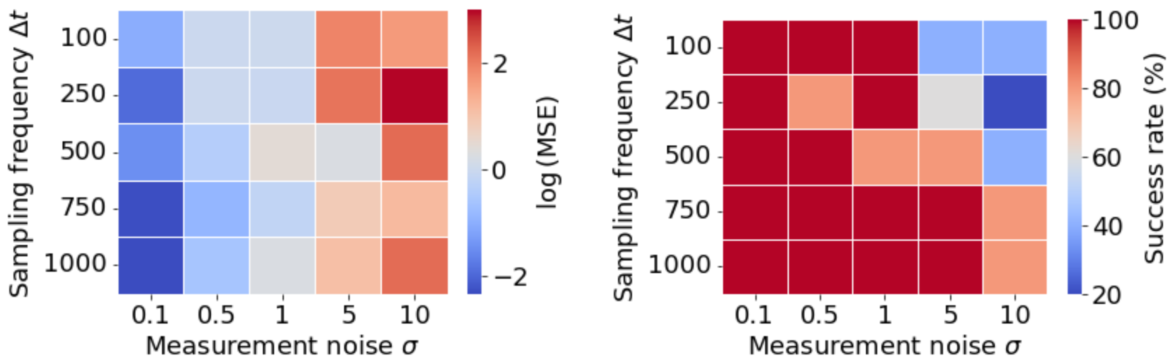

(a) (b)

Figure S.6: Sensitivity of our framework to measurement noise and sampling frequency of the signal for Lotka-Volterra system (all other configurations as in Table S.1 held constant). (a) Shows the resulting average mean squared error, and (b) shows the success rate, across five randomly sampled simulated measurement data. Note that a successful run corresponds to when the dynamics are exactly recovered and all inactive basis functions are thresholded. For high noise, low sampling rate instances, the active basis functions typically remain in the basis but often with a few other inactive basis functions with very low coefficient magnitudes (e.g., more than two orders of magnitude lower than the smallest active coefficient).

Continuously stirred tank reactor dynamical system

The basis functions used for discovering the composition dynamics were:

$$\Theta_{C_A} = \{1, C_A, T, C_A T, C_A^2, T^2, C_A^2 T, C_A T^2, 1/C_A, 1/T, e^{c/T} C_A\} \tag{S.3}$$

The basis functions used for discovering the temperature dynamics were:

$$\Theta_T = \{1, C_A, T, T_c, C_A T, C_A^2, T^2, C_A^2 T, C_A T^2, 1/C_A, 1/T, e^{c/T} C_A\} \tag{S.4}$$

The control input used to excite the composition and temperature dynamics was:

$$T_c(t) = 305 \left(1 + \frac{\sin(\pi t/5)}{125}\right) \tag{S.5}$$

A list of all other model parameters used to simulate the governing equations can be found in Table S.3.

Table S.3: Non-isothermal CSTR parameter values used for numerical experiments

**Parameter**	**Value**	**Units**
V	100	L
q	100	L min^{-1}
$C_{A,i}$	1	mol L^{-1}
ρ	1000	g L^{-1}
C	0.239	J g^{-1} K^{-1}
$-\Delta H_R$	5×10^4	J mol^{-1}
E_a/R	8750	K
k_0	7.2×10^{1}0	min^{-1}
$U\mathcal{A}$	5×10^4	J min^{-1} K^{-1}

For convergence to the true governing dynamics good initialization is often required so that the DNLP converges to or in close proximity to the global optimum. In this case, the extensive availability of reaction rate data and kinetic analyses thereof can be used to determine suitable initial guesses for parameter c. In cases when such domain knowledge is not available, statistical techniques such as the ones reported in [3] can be employed for finding good initializations. For the purposes of this study, c was initialized to -8700.

Table S.4: Configuration for CSTR numerical experiments

**System**	**Configuration**	
CSTR	Data simulation	Initial conditions: (0.28,263.5)
		Sampling frequency: 1/500
	Smoothing	$WS = 10$
		$\gamma = 10$
		$\alpha = 0.1$
	Pre-processing	Granger tests: χ^2 and F-distributions
		Granger p-value: 0.2
		OLS p-value: 0.95
		OLS % confidence: 1×10^{-5}
	Discretization	Scheme: Lagrange-Radau
		Finite elements: 200
		Collocation points: 3
		Data interpolation: Cubic spline
	Moving horizon	H: 40 (20,000 data samples)
		$\Delta\mathcal{D}$: 50 samples
		ω: 10
		Ω: 40
	Thresholding	ψ: 1

Benchmark experiments

To highlight the performance of our proposed approach relative to state of the art governing equation discovery frameworks, we perform benchmarking experiments against several versions of sparse identification of nonlinear dynamics (SINDy) [4] with their respective options (e.g., optimization algorithm, and regularization coefficient) using its Python implementation (PySINDy) [5]. Three different optimization routines are considered including: LASSO regression [6], sequential thresholded least squares (STLSQ) [4], and the more recently proposed sparse relaxed regularized regression (SR3) [7, 8]. Similar to grid search hyperparameter tuning, each combination of SINDy method (conventional SINDy [4], weak SINDY (W-SINDy) [9] and ensemble SINDy (E-SINDy) [10]) and optimization framework was evaluated for a range of thresholding tolerances and regularization penalties for each system. For LASSO regression, regularization of the form of $\lambda_{\text{LASSO}}||\Xi||_1$. For STLSQ, coefficients with $|\Xi| < \lambda_{\text{STLSQ}}$ are eliminated from the set of candidate basis functions. SR3, uses a relaxed regularization penalty of the form of $\lambda_{\text{SR3}}||\mathbf{W}||_0 + \nu_{\text{SR3}}||\Xi - \mathbf{W}||_2^2$ where $\mathbf{W}$ is a matrix of auxiliary variables. For our proposed framework, the extent of regularization of by the variability threshold ψ on the coefficient of variation for the coefficient estimates. Ensemble SINDy was run with 200 models by sub-sampling both the time series and the candidate basis function library, and the resulting models are synthesized by taking the mean. The maximum number of iterations for LASSO and SR3 were set to 2,000.

The same basis functions as in (S.1) and (S.2) were used to evaluate SINDy, with the exception of $1/\mathbf{x}$ and $e^{\mathbf{x}}$, since in the absence of nonlinear constraints to ensure that these functions remain bounded, evaluating them on the training data resulted in NaN or near infinite values which cannot be handled by the SINDy solvers considered. Furthermore, we note that for LASSO and SR3 optimizers all terms remained in the basis, albeit the majority often had very small coefficient magnitudes. Hence the equivalent zero for all dynamical system instances was set to be 5×10^{-2}, which is a conservatively large value and thus the number of terms discovered for SINDy instances is lower than for the actual equations discovered.

Table S.5: Configuration of benchmarking experiments for canonical nonlinear dynamical systems.

System	Hyperparameters for methods considered
Lotka-Volterra	$\lambda_{STLSQ} \in [1 \times 10^{-4}, 5 \times 10^{-4}, 1 \times 10^{-3}, 5 \times 10^{-3}]$
	$\lambda_{LASSO} \in [5 \times 10^{-1}, 1, 5, 10, 50, 100]$
	$\lambda_{SR3} \in [5 \times 10^{-3}, 1 \times 10^{-2}, 5 \times 10^{-2}, 1 \times 10^{-1}]$
	$\psi \in [10, 1.5, 1, 5\times^{-1}, 1\times^{-1}, 1\times^{-2}, 1\times^{-3}]$
Van der Pol	$\lambda_{STLSQ} \in [1 \times 10^{-4}, 1 \times 10^{-3}, 1 \times 10^{-2}, 1 \times 10^{-1}, 5 \times 10^{-1}]$
	$\lambda_{LASSO} \in [5 \times 10^{-3}, 1 \times 10^{-2}, 5 \times 10^{-2}, 1 \times 10^{-1}, 5 \times 10^{-1}, 1]$
	$\lambda_{SR3} \in [5 \times 10^{-3}, 1 \times 10^{-2}, 5 \times 10^{-2}, 1 \times 10^{-1}, 5 \times 10^{-1}]$
	$\psi \in [10, 1.5, 1, 5\times^{-1}, 1\times^{-1}, 1\times^{-2}, 1\times^{-3}]$
Brusselator	$\lambda_{STLSQ} \in [1 \times 10^{-4}, 1 \times 10^{-3}, 1 \times 10^{-2}, 1 \times 10^{-1}, 5 \times 10^{-1}]$
	$\lambda_{LASSO} \in [5 \times 10^{-3}, 1 \times 10^{-2}, 5 \times 10^{-2}, 1 \times 10^{-1}, 5 \times 10^{-1}, 1]$
	$\lambda_{SR3} \in [5 \times 10^{-3}, 1 \times 10^{-2}, 5 \times 10^{-2}, 1 \times 10^{-1}, 5 \times 10^{-1}]$
	$\psi \in [10, 1.5, 1, 5\times^{-1}, 1\times^{-1}, 1\times^{-2}, 1\times^{-3}]$
Lorenz	$\lambda_{STLSQ} \in [1 \times 10^{-4}, 1 \times 10^{-3}, 1 \times 10^{-2}, 1 \times 10^{-1}, 5 \times 10^{-1}]$
	$\lambda_{LASSO} \in [5 \times 10^{-3}, 1 \times 10^{-2}, 5 \times 10^{-2}, 1 \times 10^{-1}, 5 \times 10^{-1}, 1]$
	$\lambda_{SR3} \in [5 \times 10^{-3}, 1 \times 10^{-2}, 5 \times 10^{-2}, 1 \times 10^{-1}, 5 \times 10^{-1}]$
	$\psi \in [10, 1.5, 1, 5\times^{-1}, 1\times^{-1}, 1\times^{-2}, 1\times^{-3}]$

References

[1] William E Hart, Carl D Laird, Jean-Paul Watson, David L Woodruff, Gabriel A Hackebeil, Bethany L Nicholson, John D Siirola, et al. *Pyomo-Optimization Modeling in Python*, volume 67. Springer, 2017.

[2] Arne Stolbjerg Drud. CONOPTa large-scale GRG code. *ORSA Journal on Computing*, 6(2):207–216, 1994.

[3] Fernando Lejarza and Michael Baldea. Discovering governing equations via moving horizon learning: the case of reacting systems. *AIChE Journal*, page e17567, 2021.

[4] Steven L Brunton, Joshua L Proctor, and J Nathan Kutz. Discovering governing equations from data by sparse identification of nonlinear dynamical systems. *Proceedings of the National Academy of Sciences*, 113(15):3932–3937, 2016.

[5] Brian de Silva, Kathleen Champion, Markus Quade, Jean-Christophe Loiseau, J. Kutz, and Steven Brunton. Pysindy: A python package for the sparse identification of nonlinear dynamical systems from data. *Journal of Open Source Software*, 5(49):2104, 2020.

[6] Robert Tibshirani. Regression shrinkage and selection via the lasso. *Journal of the Royal Statistical Society: Series B (Statistical Methodology)*, 58(1):267–288, 1996.

[7] Peng Zheng, Travis Askham, Steven L Brunton, J Nathan Kutz, and Aleksandr Y Aravkin. A unified framework for sparse relaxed regularized regression: SR3. *IEEE Access*, 7:1404–1423, 2018.

[8] Kathleen Champion, Peng Zheng, Aleksandr Y Aravkin, Steven L Brunton, and J Nathan Kutz. A unified sparse optimization framework to learn parsimonious physics-informed models from data. *IEEE Access*, 8:169259–169271, 2020.

[9] Patrick AK Reinbold, Daniel R Gurevich, and Roman O Grigoriev. Using noisy or incomplete data to discover models of spatiotemporal dynamics. *Physical Review E*, 101(1):010203, 2020.

[10] Urban Fasel, J Nathan Kutz, Bingni W Brunton, and Steven L Brunton. Ensemble-sindy: Robust sparse model discovery in the low-data, high-noise limit, with active learning and control. *Proceedings of the Royal Society A*, 478(2260):20210904, 2022.
